# Do three years make a difference? An updated review and analysis of self-initiated expatriation

**DOI:** 10.1186/s40064-016-2991-x

**Published:** 2016-08-11

**Authors:** Diana Farcas, Marta Gonçalves

**Affiliations:** 1 Instituto Universitário de Lisboa (ISCTE-IUL), Avenida das Forças Armadas, Edifício I, 2w17, 1649-026 Lisboa, Portugal; 2 Centro de Investigação e Intervenção Social (CIS-IUL), Lisboa, Portugal

**Keywords:** Self-initiated expatriation, Integrative review, Expatriates

## Abstract

Self-initiated expatriates (SIEs) were initially described by Inkson et al. (J World Bus 32:351–368, 1997) as individuals who move abroad on their own volition, with personal funding, oriented towards development and career goals. After almost two decades of research, it is imperative to review the knowledge that has been developed and identify future areas of intervention. Doherty (Int J Manag Rev 15:447–469, 2013. doi:10.1111/ijmr.12005) initiated the review and this paper aims to update it and explore some unapproached aspects. Five different data bases were targeted and searched for peer-reviewed articles published in English, between 1997 and 2014, which recognized self-initiated expatriation as a distinguished form of mobility and used this terminology in the title and/or keywords list. A total of 94 articles met these inclusion criteria, 45 of which were published between 2012 and 2014. By systematically analyzing them, it was observed a surpassing growth in the number of published articles in the last 3 years. This signalizes an increase of the academic interest in studying the SIEs all over the world, involving bidirectional moves between developed and developing countries. The constructs identified by Doherty (2013) at the three different levels (micro, meso and macro) continued to be explored, using qualitative or quantitative approaches. Besides this, a multi-informant approach has been adopted in some studies, while others focused on concept clarification, taking into consideration some of Doherty’s (2013) suggestions for future research. Three years of research made an enormous contribution to the development of knowledge about SIEs, but some aspects can be further explored; hence they are identified and thoroughly discussed.

International mobility has received much attention in recent academic work. Several mobility patterns were identified and self-initiated expatriation is among the most studied ones, despite its relatively new recognition as an individualized form of mobility. More precisely, in 1997, Inkson et al. were the first authors who distinguished self-initiated expatriates from assigned expatriates, mainly based on the fact that they move abroad on their own volition, by personal funding, oriented towards personal goals and without any organizational support. Having this in mind, other authors (e.g. Suutari and Brewster 2000; Jokinen et al. 2008) manifested their interest in knowing more about these individuals, and research has gained pace over the last 17 years. Initially individuals studied under the banner of SIEs have included samples of university alumni and volunteers (Inkson et al. 1997), and it gradually involved more skilled/educated individuals moving on their own volition (e.g. graduate engineers: Suutari and Brewster 2000; managers and professionals: Suutari and Taka 2004; academics: Richardson 2006). After almost two decades of research, we consider that it is necessary to systematize all the developed knowledge and identify future areas of research. Therefore, we aimed to conduct an integrative literature review following Cooper’s (1998) five stage framework (Table 1).

**Table 1:** Stages of integrative review proposed by Cooper (1998)

Stage of review	Description
Formulate the research problem	Clear identification of the problem the review is addressing and its purpose
Collect data	Define inclusion criteria and search the appropriate data bases in order to collect the literature which responds to the research problem
Evaluate data	Determine if the collected literature meets the predefine inclusion criteria, and select the final literature sample
Analyze and interpret data	Examine and interpret the literature sample in such a way that it will respond to the research problem
Evaluate the state of knowledge of the phenomenon and present future directions for research	Determine all the knowledge developed around the research problem and identify what can be further explored

## Stage 1: Formulate the research problem

The research area of self-initiated expatriation is constantly evolving and in order to facilitate coherence in the development of future research, literature reviews should be conducted. They are very useful in clearly presenting existing research and identifying possible areas of future intervention. Doherty (2013) initiated this literature review, focusing on the research conducted between 1997 and 2011. However, due to the constant evolution of self-initiated expatriation research and the increase in the number of people living abroad, we consider that it is pertinent to conduct a new literature review which may update the previously conducted one. More specifically, the aims of our integrative literature review are two folded. First, we aim to compare and contrast the systematized research conducted between 1997 and 2011 with the research which has been carried out over the past 3 years (2012–2014). Second, regarding future areas of research, we aim to determine to what extend Doherty’s (2013) suggestions were met during the second research period and identify what else can be researched.

## Stage 2: Collect data

In order to reach the proposed aims, data were gathered by a series of searches undertaken using the following databases: PsycINFO, Web of Science, Emerald, ABI inform (Proquest) and Business Complete. “Self-initiated expatriat” was used as a keyword in the topic field, accompanied by the wildcard ‘*’, in order to assure that all the possible combinations of the keyword (e.g. self-initiated expatriates, self-initiated expatriation) were obtained. Once extracted, overlapping articles among the different databases were excluded, and the remaining articles were screened in order to guarantee that they specifically used the terminology of “self-initiated expatriation” as a distinguished form of mobility, appearing in the title and/or keywords list. At the same time, we checked for the document type, restricting it to peer-reviewed articles, in order to enhance quality control. English was the chosen language for the articles published between 1997 and 2014. We chose to limit the data of publication to these 17 years, in order to gather the articles reviewed by Doherty (2013) from 1997 until 2011 and the new ones published between 2012 and 2014.

## Stage 3: Evaluate data

For the 1997–2011 period, the retrieved articles were doubled checked in order to make sure that they corresponded to the ones found by Doherty (2013). In addition, for the 2012–2014 period, the articles were screened according to the predefined inclusion/exclusion criteria. As a result of this, twelve publications were excluded (three dissertations, one guest editorial article, three articles which did not address self-initiated expatriates independently, one conference abstract, two articles written in Portuguese, a corrigendum paper and an article which was published twice, in a special and normal issue). The final list offers a total of 94 articles, 45 of which were published between 2012 and 2014.

## Stage 4: Analyze and interpret data

Data were analyzed using Doherty’s (2013) method of systematization; hence the selected articles were reviewed under four categories: study focus, methods, findings/stated contributions and identified gaps/areas for future research (“Appendix” presents a summary of this review).

## Comparing the research conducted between 1997 and 2011 versus 2012–2014

The reviewed articles were compared in terms of the research context, methodological approach and studied variables/constructs. In addition, since Doherty (2013) left some suggestions for future research, we explored the extent to which they were met during the second research period (2012–2014).

### Research context

The research on self-initiated expatriation started in Australia and New Zealand (Inkson et al. 1997), which were either home or host countries. In other words, the sample of this study comprised SIEs who relocated to Australia/New Zealand but it also included SIEs from Australia and New Zealand who relocated to other countries, such as the United Kingdom. Subsequently, research on self-initiated expatriation extended to some other host countries in Europe (e.g. France and Germany: Crowley-Henry 2007) and a limited number of countries in the Middle East (e.g. Saudi Arabia: Bhuian et al. 2001; Bozionelos 2009) and Asia (e.g. Japan: Peltokorpi 2008). During the period of 2012–2014, an increased number of studies have ranged across SIEs who relocated to Asia (e.g. China: Lauring and Selmer 2014; Muir et al. 2014; Selmer and Lauring 2014b; South Korea: Froese 2012; Macau: Lo et al. 2012; Japan: Froese and Peltokorpi 2013), Middle East (Saudi Arabia: Alshammari 2012; Qatar: Scurry et al. 2013) and Europe (Denmark: Bjerregaard 2014; Germany: Cao et al. 2013). In addition, a limited number of studies were conducted in North and South American countries (e.g. USA: Farndale et al. 2014; Canada: Richardson and McKenna 2014; Brasil: von Borell de Araujo et al. 2014). In terms of the studied SIEs’ move between home and host country, it can be observed that it mostly occurs between developed countries (e.g. New Zealand-Belgium: Ellis 2012), followed by developing to developed ones (e.g. China-Germany: Cao et al. 2013) and very few take place between developed to developing countries (e.g. USA-Brazil: von Borell de Araujo et al. 2014).

### Methodological approaches

Both qualitative and quantitative approaches are methodological strategies used to study the self-initiated expatriation phenomenon. During the first period of time (1997–2011), studies mainly targeted the individuals who undertook this mobility pattern, while over the past 3 years (2012–2014) a multi-informant perspective was adopted, involving the perspective of the host country nationals (e.g. Ellis 2012), SIEs’ supervisors (e.g. Showail et al. 2013) or spouses (e.g. Bjerregaard 2014), as a complement to SIEs’ view regarding a determinant issue. The number of quantitative and qualitative studies which were conducted is almost equal during both time periods. However, longitudinal studies prevailed only in the first period (e.g. Hudson and Inkson 2006).

An inequality can be observed in the number of studies where a literature review was conducted. During the period of 2012–2014, 10 literature reviews were conducted, which corresponds to more than twice the number of literature reviews conducted in the previous period. It is important to mention that although the methodology coincided, the purpose and ultimate result of the literature reviews differed.

For example, two of the four literature reviews conducted during the first period, focused on the theoretical exploration of gender issues in SIE (Tharenou 2010) and HR implications of SIEs’ adjustment (Howe-Walsh and Schyns 2010), while the other two reviewed the existing literature with the aim of identifying alternative forms of international workers (McKenna and Richardson 2007) and developing a conceptual understanding of SIEs’ careers (Tams and Arthur 2007). These two topics along with the mere systematization of the conducted research were further explored in the ten literature reviews conducted during the past three years (2012–2014).

More precisely, the literature reviews conducted by Cao et al. (2012) and Whitman and Isakovic (2012) focused on developing a conceptual framework with propositions predicting career success for SIEs and the influence of personality and stress management on SIEs’ and AEs’ international experience success. This comparison between SIEs, AEs and other forms of mobility, along with the conceptual clarification of what it means to be a SIE was explored in six more reviews. Cerdin and Selmer (2014) provided a definition of who is a SIE based on four mutually satisfied criteria: self-initiated international relocation, regular employment, intentions of a temporary stay and skilled/professional qualifications. In addition, Tharenou (2013) identified several conditions where SIEs can be a suitable replacement of AEs (e.g. technical and middle/lower management positions), while Shaffer et al. (2012), Al Ariss and Crowley-Henry (2013), Doherty et al. (2013) elaborated a profile of SIEs based on different aspects (e.g. country of origin, gender, period of international mobility) which were contrasted with migrants, AEs, short term assignees, flexpatriates, international students and international business travelers. In order to simplify the reading of the criteria distinguishing the different mobility groups, Andresen et al. (2014) proposed a decision tree.

### Studied variables/constructs

According to Doherty (2013), the produced knowledge about SIEs can be organized at three levels of analysis: micro, meso and macro. At the micro level, the variables involved concern the individual characteristics and experiences of SIEs (e.g. demography, motivational drivers, individual characteristics, adjustment, career anchors), while the meso-level variables involve work-related experiences of SIEs (e.g. performance measures, career development, organizational context). The third level of analysis takes into consideration the home and host context, focusing on variables associated with human capital and the talent flow magnitude.

By taking this information into consideration, first we present some empirical studies which compared SIEs to AEs in terms of variables situated at the three levels of analysis, with the micro and meso levels prevailing. Afterwards, the empirical studies which focused predominantly or solely on SIEs will be described in terms of the studied variables and encountered results at each one of the three levels.

It is important to mention that all the empirical studies conducted between 1997 and 2014, which were specifically targeted at SIEs and AEs are systematized in Table 2. Most of these studies (8/11) were conducted during the first period of time (1997–2011) and the variable/constructs explored are similar to the ones studied in the second period of time (2012–2014). Regarding the encountered results, several similarities and differences were encountered between SIEs and AEs on each one of the nine explored variables.

**Table 2:** Results from studies comparing SIEs and AEs

Variables	Differences between the two type of expatriates		Similarities between the two types of expatriates	Authors
	Self-inititated expatriates (SIEs)	Assigned expatriates (AEs)		
Motivational drivers	Interest in internationalism and poor employment situation	Employer initiative		Suutari and Brewster (2000)
	Location and host country reputation	Career factors		Doherty et al. (2011)
Geographical mobility	More likely to move from peripheral to economically advanced countries	Move more easily to peripheral countries		Peiperl et al. (2014)
	Move where conditions offer greater economic prospects	Move to less developed countries and support the company subsidiary there		
Demographics and individual characteristics	Slightly younger, more females and singles, accompanied with spouses working abroad	Older, more males, married, accompanied with spouses not working abroad		Suutari and Brewster (2000), Peiperl et al. (2014)
	More proficient in host country language	Less proficient in host country language	No significant difference were found in age, gender, marital status or education	Froese and Peltokorpi (2013)
	SIE spend more time in host country	Have more international experience in working abroad	High open-mindedness, cultural empathy and social initiative	
Career	Lower levels of knowing whom	Higher levels of knowing whom	High levels of knowing how and knowing why	Jokinen et al. (2008)
	More stable career orientation/personal investment in career and career progression sustained over time	Career orientation decreases with age		Biemann and Andresen (2010)
	Security anchor	Internationalism anchor	Lifestyle anchor	Cerdin and Le Pargneux (2010)
	Boundaryless career	Protean		Inkson et al. (1997)
Employer, job and task variables	More often employed at lower organizational levels	Occupy high organizational level/managerial positions		Suutari and Brewster (2000), Froese and Peltokorpi (2013)
	Employment organizations are international or foreign private companies	Tend to work in home country companies and their respective subsidiaries		
	Undertake relatively unskilled, casual roles, often below their capabilities	Roles are broader and more challenging, according to their capabilities		Inkson et al. (1997)
	Higher organizational mobility and intention to change organization	Lower organizational mobility and intention to change organization		Biemann and Andresen (2010)
	Less satisfaction with job	Higher levels of job satisfaction		Froese and Peltokorpi (2013)
Compensation	High variations in net salary levels	Less variation in salary		Suutari and Brewster (2000)
	Less common or inexistent additional competitive compensation packages (assignment insurance, overseas premiums, house and education allowances)	Very common additional competitive compensation packages (assignment insurance, overseas premiums, house and education allowances)		
Coping strategies	Less critical and more willing to emulate typical host country behaviors for resolving problems related to adaptation to the country	Negative interpretation of the entire cultural system and dissatisfied		von Borell de Araujo et al. (2014)
Adjustment	Interact with local populations, understand better the language and culture, adjusting more easily	Do not interact as much with host country nationals, and have more difficulties to adjust		Sargent (2002), Peltokorpi and Froese (2009), Froese and Peltokorpi (2013)
			Challenges to adjustment related to obtaining a visa, renting a house, contracting for utilities and paying taxes	von Borell de Araujo et al. (2014)
Repatriation	No repatriation agreement is made prior to departure, and are more willingly to accept another working period abroad	Usually move abroad with a definite timeframe and repatriation agreement		Suutari and Brewster (2000)

#### Micro, meso and macro level research comparing SIEs and AEs

The similarities between SIEs and AEs are focused on individual characteristics, career and adjustment. In terms of individual characteristics, Froese and Peltokorpi (2013) found out that the studied expatriates (SIEs and AEs) who were living and working in Tokyo, scored high on the multicultural personality questionnaire, in terms of open-mindedness, cultural empathy and social initiative. At the same time, the career capital of the Finish expatriates (SIEs and AEs) studied by Jokinen et al. (2008) was similar in terms of the knowing how (explicit work-related knowledge required for performance) and knowing why (motivation and identification with the work world) dimensions, while the lifestyle anchor was the most valued one by the French expatriates who participated in the study conducted by Cerdin and Le Pargneux (2010). Additionally, the SIEs and AEs in Brazil (von Borell de Araujo et al. 2014) faced similar challenges to adjustment, concerning legal (obtaining visa, paying taxes) and practical aspects (renting a house, contracting for utilities).

However, SIEs differ from AEs in the way they overcome these challenges and adjust to the host country. More precisely, SIEs are less critical than AEs, and more willing to emulate typical host country behaviors for resolving adjustment problems (von Borell de Araujo et al. 2014). This might be one of the reasons why several studies consider SIEs to adjust more easily, along with the fact that they are more predisposed to interact with local populations and understand better the host country’s language and culture (Sargent 2002; Peltokorpi and Froese 2009; Froese and Peltokorpi 2013). The motivational driver can be another reason for SIEs’ adjustment being easier than AEs’, since SIEs move abroad on their own volition, while AEs are chosen by the employer (Suutari and Brewster 2000). Most often, SIEs choose the host country, and are more likely to move from peripheral to economically advanced countries, where conditions offer greater economic prospects. This does not happen with AEs, who can move to less developed countries, supporting the company subsidiary which is located there (Peiperl et al. 2014; Froese and Peltokorpi 2013). Therefore, AEs are more likely to occupy high organizational level/managerial positions and have broader and more challenging (i.e. high level responsibilities) roles than SIEs, with stimulating salary and compensation packages (Inkson et al. 1997; Suutari and Brewster 2000). In addition, AEs tend to have more international experience of working abroad than SIEs (Froese and Peltokorpi 2013). In comparison to SIEs, they also tend to be older, predominately male, married and usually accompanied by spouses who do not work abroad (Suutari and Brewster 2000; Peiperl et al. 2014). It is important to mention that these demographic differences were not found in the study conducted by Froese and Peltokorpi (2013); hence they should be carefully interpreted.

#### Micro level research

The first variables explored at an individual level reflect the interest of identifying who the SIEs are and what makes them move abroad. The individual characteristics of SIEs were exclusively explored during the first period of time, suggesting that they can be characterized as self-reliant, autonomous and individualistic (Inkson et al. 1997; Sullivan and Arthur 2006). Additionally, six studies conducted during the first period 1997–2011, identified several reasons which make SIEs undertake an international mobility (Table 3).

**Table 3:** Comparing micro level research between two research periods (1997–2011 vs. 2012–2014)

Period	
1997–2011	2012–2014
*Motivational drivers*	
A desire for exploration and excitement, a positive predisposition to the experience prompted by family and social connections, to escape from a current way of life or job situation (Inkson et al. 1997; Inkson and Myers 2003) Economic factors, better opportunities/income, career-vocational opportunities, family, life-style, cultural distance and political environment. Pull Factors: life style and family considerations; Push Factors: career, culture and economics (Jackson et al. 2005) Chance rather than a result of a specific plan, desire for adventure, life change and benefit to the family (Richardson and Mallon 2005) Several sub-motives underlie the motivation to go abroad, related to career, cultural/travel opportunities, economic/personal relationships. These vary with gender, location and life stage (Thorn 2009) The motives to expatriate (adventure/travel, career, family, financial incentives and life change/escape) differ in terms of acquired personal characteristics: marital status, nationality, previous expatriate experience and seniority (Selmer and Lauring 2011b)	The advances in education and careers, escape mandate of military service (Beitin 2012) A desire for international experience, attractive job conditions, family ties, and poor labor markets in home countries (Froese 2012) Motivational drivers were grouped in four sets of reasons: refugee, mercenary, explorer and architect (Selmer and Lauring 2012) Cultural and travel opportunities, career, economics, affiliations, political environment, and quality of life (Thorn 2013) Tourism-oriented and work-related motivations were stronger among academic SIEs who are younger, non-married, non-EU and with short experience. Non-EU SIEs arriving in the EU have stronger financial and seeking motivations (Lauring et al. 2014)
*Demographics (gender, marital status, age)*	
Women’s willingness to go abroad is more affected by family/relationships than men’s (Myers and Pringle 2005; Tharenou 2008) Women chose less risky environments, which can offer them international career opportunities and more career benefit than men (Myers and Pringle 2005) Women are less motivated to go abroad by financial gain and life change (Selmer and Lauring 2010) Positive relationship between marital status and work effectiveness/performance is not moderated by gender (Selmer and Lauring 2011a)	Female SIEs have better job performance than male SIEs (Lauring and Selmer 2014) Married SIEs have better time to proficiency and job performance than unmarried SIEs (Lauring and Selmer 2014)
*Individual characteristics*	
Self-reliant, autonomous, exhibiting diffuse individual developmental goals and valuing the cultural experience and opportunity for personal learning, as opposed to purely work experiences (Inkson et al. 1997) Individualistic, non-conformist, self-reliant, self-directed and proactive, operating with a degree of personal agency and giving personal motives precedence in determining their psychological and physical mobility (Sullivan and Arthur 2006)	
*Career*	
	The metaphor “river” is proposed to describe career development (Crowley-Henry 2012) Career agency is impacted by both individual (e.g. personal control, proactivity, self-determination) and contextual factors, which provide support for Tams and Arthur’s (2010) six dimensions of career agency (Guo et al. 2013) Careerist attitude and career fit explain international mobility success, while the influence of protean and boundaryless career attitude is not very clear. Careerist orientation is the individual career characteristic which better explains international mobility success (Cerdin and Le Pargneux 2014) Four career patterns are identified: reinventors, reinvigorators, reversers and rejecters (Muir et al. 2014)
*Adjustment*	
Language proficiency, personality traits, cultural empathy and type of expatriation experience (SIE vs. AE) have a positive effect on work and non-work adjustment; SIEs adjust better than AEs (Peltokorpi 2008) Positive framing and proactive socialization enable more effective coping and adjustment (Fu et al. 2005)	Previous international experience and marital status have no influence on adjustment (Alshammari 2012) Previous overseas experience has a positive relationship with SIEs’ adjustment, while culture novelty has a negative one. Contrary to what was predicted, foreign language ability was not positively related to adjustment (Isakovic and Whitman 2013) Positive cross-cultural adjustment mediates the positive relations between protean career attitude and SIEs’ experienced outcomes: career satisfaction, intentions to stay in the host country and life satisfaction (Cao et al. 2013) Beneficial associations between positive affectivity and adjustment (Selmer and Lauring 2014a) Adult third-culture kids have a greater extent of general adjustment, but not interaction or work adjustment, when compared with adult mono-culture kids (Selmer and Lauring 2014b)
*Relationship with home and host country/repatriation*	
The propensity of moving was explored through allegiance, a dynamic and fluid bond that influences both the desire to remain in the host country and the desire to return to home country. Family and social connections have a great impact on the intention to stay or return (Richardson and McKenna 2006; Schoepp and Forstenlechner 2010) Weak host country pull and strong home country pull, along with shocks motivate repatriation (Tharenou and Caulfield 2010) After repatriation, adjustment to work is a stressful experience, since SIEs do not return to a role within an organization and have to reacquire local experience and rebuild networks (Begley et al. 2008)	Relationship with home and host country are fluid and subject to change due to adjustment and ease of communication (Beitin 2012) Desire for cultural and travel opportunities was the best predictor of cessation of mobility and development level in the host country. Career motives predicted duration of mobility and cultural difference of the destination (Thorn et al. 2013)

This variable continued to receive great attention in the second time period (2012–2014), with a particular interest in directly identifying the key stimuli factors in moving abroad for specific populations, determining the influence of motivational factors on other variables and testing the previously encountered results.

For example, Froese (2012) identified that the motivational drivers of SIEs in South Korea consist of poor labor markets in home countries versus attractive job conditions in the host countries, and a desire for international experience. Similarly, the Syrian SIEs studied by Beitin (2012) identified motivational factors related with the home (escape from the military service mandate) and host countries (possibility of advancing in education and careers). This similarity in the motivational drivers among two different populations sparked some interest in identifying if there is a relationship between motives, mobility patterns and demographics. Thorn et al. (2013) grouped the identified motives in six different categories: cultural and travel opportunities, career, economics, affiliations, political environment and quality of life. The influence of these motives on the mobility pattern showed that the desire for cultural and travel opportunities is the best predictor for mobility cessation and developmental level in the host country, while career motives predicted mobility duration.

Lauring et al. (2014) proposed another grouping order for the motivational drivers, by taking into consideration the extent to which they relate to work (career and financial reasons) or are more tourism-oriented (seeking and escape reasons). These latter motivations are strongest among SIE academics who are young, non-married and originally from a non-EU country. The SIEs from a non-EU country moving to the EU are motivated by financial and seeking motivations. According to Selmer and Lauring (2012), these motivations would fit under the refugee or mercenary categorization of motivational drivers, based on the behavioral intentions and outcome control matrix. This matrix takes into consideration affective and evaluative behavioral intentions along with the easiness or difficulty of SIEs’ outcome control. Therefore, by combining these four dimensions, the equivalent number of categories emerges, indicating that SIEs can be classified as a refugee (motivated by life change and escape reasons), a mercenary (motivated by financial incentives), an explorer (motivated by a desire for adventure and travelling) or an architect (motivated by career considerations).

This categorization was initially proposed by Richardson and McKenna (2002), and its empirical proof and effect on work outcomes (work performance, work effectiveness and job satisfaction) was tested by Selmer and Lauring (2012). The categorization of SIEs’ reasons to expatriate was partially confirmed, since the participants in this study considered that what influenced their decision to expatriate was mainly the desire for adventure, financial gains and career opportunities. Therefore, they did not identify as much with the refugee reason which refers to the escape from previous life situations. However, this was the best predictor for work outcomes. A strong negative association was found between the refugee reasons to expatriate and the three work outcomes, namely work performance, work effectiveness and job satisfaction. Selmer and Lauring (2012) considered this a striking finding, because one may speculate that refugee reasons to relocate result in negative work outcomes. Nonetheless, they argue that the interpretation of these results may not be straightforward, since the studied group of SIEs was not homogeneous and SIEs who relocated from developing countries to developed ones might have escaped from undesirable living conditions in their home countries; hence relocated more by necessity than by choice. Consequently, these SIEs may have experienced discrimination in the organizational context of developed countries, due to ethnic traits such as language, religion or clothing habits, just as other studies pointed out (e.g. Al Ariss 2010; Al Ariss and Özbilgin 2010). Regarding the mercenary reasons to expatriate, Selmer and Lauring (2012) did not identify any relations with the work outcomes. This may indicate that financial reasons are not as important for SIEs as they might be for AEs (Richardson and Mallon 2005). On the other hand, just as predicted, the explorer reasons to expatriate are strongly related to job satisfaction, while architect reasons facilitate work performance and work effectiveness.

Besides motives’ effect on work outcomes, demographical variables were also explored during both time periods. More specifically, the relationship between gender, marital status and job performance was studied during both time periods and similar results were encountered. Selmer and Lauring (2011a) along with Lauring and Selmer (2014) identified that married SIEs have better job performance than unmarried SIEs. However, the results found by these authors differ regarding gender. On the one hand, Selmer and Lauring (2011a) identified that the positive relationship between marital status and job performance was not moderated by gender. Also, when gender was entered alone in the moderation model, it did not result in any significant effect on the job performance. On the other hand, Lauring and Selmer (2014) determined that female SIEs have better job performance than male SIEs. Also, married SIEs have better job performance and are more satisfied with their job than unmarried SIEs.

The influence of gender was further explored exclusively during the first time period, through an association with the motivational drivers. The results indicate that when comparing men with women in terms of their willingness to go abroad, women are more affected by family/relationships and less motivated by financial gain and life change (Myers and Pringle 2005; Tharenou 2008; Selmer and Lauring 2010). In addition, women chose less risky and more secure environments, which offer them international career opportunities (Myers and Pringle 2005).

During the second period of time, researchers devoted a lot of attention to SIEs’ careers, with the aim of characterizing careers and identifying what type of career contributes the most to a successful international experience. In order to characterize the SIEs’ careers, four different patterns were identified and a new metaphor was proposed to describe career development. The chosen metaphor was a river, referring to “high or low starts, different tributaries (opportunities and challenges) flowing in and out of the career river at different stages; some rivers growing large, while others fading away and perhaps then following and growing again along a different path” (Crowley-Henry 2012, p. 134). In addition, the four patterns identified by Muir et al. (2014) were reinventors (reinvent self and career), reinvigorators (adapt the existent possessed skills to the new working environment), reversers (unable to pursue the desired career path, since it has stalled or gone backwards) and rejecters (overwhelmed by the challenges faced in the work context). In order to identify what type of career contributes the most to a successful international experience, Cerdin and Le Pargneux (2014) used three variables that characterize an individual’s internal career: protean career attitude, boundaryless career attitude and careerist orientation. Results indicated that the success of international mobility, in terms of job satisfaction, career satisfaction and intentions to leave, was best explained by a careerist orientation.

However, besides career orientation, there are other variables which can predict SIEs’ international mobility success, such as cross-cultural adjustment. During both time periods, researchers attempted to determine the factors which positively and negatively contributed to SIEs’ adjustment in the host country. The results are inconclusive with respect to the influence of previous international experience and language proficiency, since either positive, negative or no effects on SIEs’ adjustment were found (Peltokorpi 2008; Alshammari 2012; Isakovic and Whitman 2013). But, it is well known that culture novelty (unfamiliar host country culture or a high degree of difference between the host and home country’s cultures) influences SIEs’ adjustment negatively, while the personality trait, cultural empathy has a positive influence (Peltokorpi 2008; Isakovic and Whitman 2013).

Mal-adjustment or even lack of adjustment have been reported as prompting factors of early repatriation among AEs (Bhaskar-Shrinivas et al. 2005), while family and social connections have a great impact on SIEs’ intentions to stay or return (Richardson and McKenna 2006; Schoepp and Forstenlechner 2010). In addition, by relating the motivational drivers to SIEs’ repatriation, Thorn et al. (2013) found out that the desire for culture and travel opportunities was the best predictor of mobility cessation, while career motives predicted duration of mobility and cultural distance between home and host country.

#### Meso level research

Organizational environments have grown accustomed to receiving AEs from different countries, who are sent by the employers to accomplish a job or organizational goal, during a specific period of time. However, due to the rapid pace of globalization, an increase in the range of global populations could be observed. Therefore, besides the corporate expatriates (AEs) there are other types of mobility patterns involved in the global labor market, such as SIEs. Much of the research conducted during the first period (1997–2011), focused on SIEs’ behavior in the corporate environment, exploring aspects related to the recruitment process, talent management and repatriation (Table 4).

**Table 4:** Comparing meso level research between two research periods (1997–2011 vs. 2012–2014)

Period	
1997–2011	2012–2014
*Recruitment*	
A more holistic approach should be adopted in the recruitment process, incorporating realistic job previews and living conditions (Richardson et al. 2008) Proactive socialization and positive framing (Fu et al. 2005)	
*Talent management*	
Income is a significant motivator to work in the international context (Bhuian et al. 2001) Job satisfaction and job variety are predictors of organizational commitment, while job autonomy is negatively related to it (Bhuian et al. 2001) Peer support is related to job satisfaction, while mentor support relates to job satisfaction and turnover intentions (Bozionelos 2009) Antecedents of underemployment: lack of job autonomy, job suitability, job variety and fit to psychological contract. Consequences of underemployment: negative effect on job satisfaction, leading to higher levels of work alienation and lower career satisfaction (Lee 2005)	Performance management (PM) preferences of SIEs: goal-setting, performance measurement and appraisal, and a performance-based pay component. The Belgians preferences for PM incorporate professional and distant relationship (Ellis 2012) On average, SIEs in the public sector present a higher degree of performance and effectiveness than SIEs in the private sector. Contrary to what was predicted, this doesn’t happen with job satisfaction. For SIEs in the private sector vs. the public sector, there is a stronger positive association between creativity and performance, creativity and effectiveness, but not between creativity and job satisfaction (Lauring and Selmer 2013) The relationship between role ambiguity and job performance was significant. This relationship was mediated by organizational identification and moderated by information seeking and perceived organizational support (Showail et al. 2013) Work adjustment is explained by self-efficacy beliefs among global employees. Job satisfaction is explained by job factors (role discretion and role conflict) and organizational or job context factors (supervisory support and perceived organizational support). Both work role adjustment and job satisfaction are not influenced by whether or not the global employee is company assigned or self-initiated. (Supangco and Mayhofer 2014)
*Repatriation*	
Family and social relationships have a significant impact on the intention to stay in the host country or return to the home country, hence a possible management strategy could involve providing return trips (Richardson and McKenna 2006) Underemployment in the host country and not returning to a role within an organization in the home country, increases the level of stress (Begley et al. 2008) Host country push–pull factors, home country pull factors and shocks can contribute to repatriation (Tharenou and Caulfield 2010)	A direct positive effect between perceived organizational support (POS) and intention to stay was found. However, there was a significant negative indirect effect between POS and intention to stay when the career network of home country nationals was large. POS has a positive effect on SIEs’ career satisfaction and intention to stay in the host country (Cao et al. 2014)

During the second period, talent management and repatriation continued to receive much of researchers’ attention. In terms of the strategic management of SIEs, new insights were proposed regarding their performance management preferences, work adjustment and effectiveness. When compared with the host country nationals, whose preferences incorporate professional and distant relationships, SIEs’ performance management preferences in Belgium include goal-setting, performance measurement and appraisal, as well as a performance-based pay component (Ellis 2012). These results reinforce the ones encountered in the first period, in terms of the income motivator to work (Bhuian et al. 2001). At the same time, the preference for goal-setting is further explained by taking into consideration the negative relationship which exists between role ambiguity and job performance. In fact, SIEs prefer to know exactly what behavior is expected from them; otherwise their performance is not at its highest potential. Nonetheless, the relationship between role ambiguity and job performance is mediated by organizational identification, and moderated by information seeking and perceived organizational support (Showail et al. 2013).

Perceived organizational support along with supervisory support, role discretion and role conflict, were the job context factors which explained SIEs’ job satisfaction better than self-efficacy belief or status of expatriation (Supangco and Mayhofer 2014). Moreover, when comparing SIEs in the public sector with SIEs in the private sector, it was observed that SIEs in the public sector presented a higher degree of performance and effectiveness than SIEs in the private sector (Lauring and Selmer 2013).

In terms of repatriation intentions, intentions to stay in the host country were explored in an innovative way, by directly linking them with organizational aspects. In other words, while in the first period (1997–2011), family, social relations and host/home country push–pull factors, were explored, Cao et al. (2014) focused on perceived organizational support. They determined that the intention to stay in the host country was positively related to perceived organizational support. However, the inverse relationship occurred when SIEs had a large career network of home country nationals in the host country. This result is congruent with the findings of Richardson and McKenna (2006), lending support to the claim that social networks play an important role in SIEs’ international relocation experiences. In addition, several factors positively predict for pre-migration adaptation, such as previous international work experience, perceived organizational prestige, satisfaction with time, information and assistance to prepare for relocation and quality contact with host country nationals during recruitment (Yijälä et al. 2012).

#### Macro level research

During the first research period (1997–2011), SIEs emerged and were soon recognized as a potential resource in the rapidly growing global economy. Carr et al. (2005), pointed out the creation of virtual networks where SIEs could share their experience and expertise with home country nationals, as one way of SIEs benefiting organizations and home countries. In addition, SIEs were considered to be a valuable asset for organizations since they are present and contribute to the global economic workforce, possessing skills, knowledge and abilities which position them advantageously in international employment contexts. This is greatly due to the fact that they are intrinsically motivated to move abroad without any organizational support, which distinguishes them from the AEs. Several studies compared SIEs to AEs, and although SIEs are seen as a potential asset for organizations, there is lack of empirical data on the flow, scope and magnitude of self-initiated expatriation. In other words, SIEs are difficult to locate and there is little evidence of how organizations acquire SIEs and which skills contribute the most to the organizational context. Therefore, it was suggested that a contribution to the macro level research on SIEs would be the exploration of the individual-organizational relationship.

Much of the macro level research conducted during the second research period (2012–2014) focused on filling some of the gaps identified in the previous research period. Specifically, Tharenou (2013) focused on the identification of the situations where SIEs could replace AEs (e.g. roles requiring cross-cultural and host location specific competencies), while the home and host country relationships were a priority in several studies. For example Lo et al. (2012), identified that host country organization embeddedness mediates the relationship between home country community embeddedness and SIEs’ turnover intentions. In addition, Yijälä et al. (2012) determined that SIEs’ European identification mediated the relationship between previous international work experience and organizational identification. An increase in the cross-border mobility of highly skilled individuals is observed during this period, but precise data on the intra-European mobility are limited because these assignments do not necessarily require a work permit. Therefore, just as pointed out by Doherty (2013), the development of standardized instruments to measure the scope and magnitude of self-initiated expatriation, remains a suggestions for future research.

### Suggestions for future research

After analyzing all the articles published during the first research period (1997–2011), Doherty (2013) identified some aspects which could be explored in upcoming studies. During the second research period (2012–2014), almost half of these suggestions were actually taken into consideration, as it can be observed in Table 5.

**Table 5:** The proposed future research suggestions by Doherty (2013) and the extent they were met during the 2012–2014 period

Doherty’s (2013) proposed future research suggestion	Met/unmet during the 2012–2014 research period	Example of conducted study during the 2012–2014 research period
“Explore the shifts that SIEs may make between self-initiated, company supported and migrant status.” (p. 450)	×	
“The gendered nature of some facets of the experience is an important issue worthy of further study.” (p. 451)	×	
“There is little evidence of how organizations acquire SIEs or the extent to which the skills contributed by SIEs are matched to the organizational context.” (p. 456)	×	
“Further research is needed in order to gauge the magnitude of the population to provide evidence of the scale of SIEs as a potential global resource.” (p. 458)	×	
“Further research could explore issues such as intra and inter home and host country comparisons affecting the intention to become SIEs, exploration of the factors that affect intended and actual repatriation behavior and the many facets of employment such as job satisfaction, organizational identification and commitment.” (p. 458)	√	Cao et al. (2014)
“Further research could usefully be done to validate constructs such as career anchors of SIEs.” (p. 458)	×	
“There is a need to address research questions relating to the utility of SIEs to corporations and meso-level issues about the employee-employer relationship.” (p. 458)	√	Tharenou (2013)
“Further research could poll managers’ perceptions of SIEs to provide data on how SIEs are perceived within the organizational context.” (p. 458)	√	Showail et al. (2013)
“There is a need to explore how individual-level variables can relate to the organizational level, further researching, for example, how the adjustment patterns among SIEs can connect to organizational performance.” (p. 459)	×	
“A further step in theoretical development is required to demonstrate whether and how the individual level career capital of SIEs can contribute to an organizational-level competitive advantage.” (p. 459)	√	Cerdin and Le Pargneux (2014)

As suggested by Doherty, Cao et al. (2014) explored some facets of employment (e.g. career satisfaction, perceived organizational support) associated with SIEs’ intention to stay in the host country. Regarding the utility of SIEs to corporations and meso-level issues, Tharenou (2013) explored the situations where SIEs could replace AEs. As a complementary perspective, managers’ perceptions were included in a study that tested the relation between role ambiguity, organization identification and job performance (Showail et al. 2013). The influence of individual career capital on SIEs’ international mobility success was explored in a study conducted by Cerdin and Le Pargneux (2014).

## Stage 5: Evaluate the state of knowledge of the phenomenon and present future directions for research

By directly answering the question presented in the title of this article, it can be affirmed that 3 years make a lot of difference. While updating and synthesizing the literature review of the published articles on the self-initiated expatriation topic from 2012 until the end of 2014, we observed a massive interest and exponential growth in this topic. This bourgeoning interest in the topic of self-initiated expatriation can be related to the statistical data provided by the UN Department of Economic and Social Affairs’ Population Division (2011), which accounts for 214 million individuals living and working outside their country of origin in 2010. This represents an increase of 58 million since 1990 and about 3 percent of the total world population. At the same time, SIEs are considered to be valuable individuals for international human resource management, benefiting organizations and economies (Dickmann and Baruch 2011). Therefore, academic scholars, businesses and policy-makers have been manifesting their interest in knowing more about these individuals living and working abroad on their own volition.

This affirmation is corroborated by the 45 peer-reviewed published articles over a period of 3 years (2012–2014), which is almost equal to the number of published works (49) during the preceding 14 years (1997–2011). By comparing the two research periods (1997–2011 vs. 2012–2014) in terms of the research context, we observed an expansion of the countries where research has been conducted. In other words, the research on SIEs started in Australia and New Zealand, involving some European countries and a limited number of countries from the Middle East and Asia. During the second research period, an effort was made to fill in the gap, by expanding the research on SIEs to the countries in which self-initiated expatiation was limitedly explored. Therefore, much of the research conducted during 2012–2014 ranged across SIEs in Asia, Middle East and Europe, while a limited number of studies were also conducted in North and South American countries. By analyzing the SIEs’ move between home and host countries, it could be observed that it mainly occurred between developed countries, followed by developing to developed ones, while very few took place from developed to developing countries. Future research could explore SIEs’ move between developed and developing countries, such as, but not limited to, the move from Portugal to Brazil or Angola, since the statistical data indicate that these two host countries are among the top destinations of the contemporary wave of Portuguese migration. In addition, in terms of cultural distance and adjustment these two countries are of potential interest since they were Portuguese colonies, and some cultural similarities might be explored in order to determine if they facilitate the SIEs’ adjustment process. More precisely, the results of several studies point out host country language proficiency as a predictor of SIEs’ adjustment. By taking into consideration that Portuguese is the official language of Portugal and Brazil, then an assumption could be made and further explored concerning a Portuguese SIE’ s facilitated adjustment process.

By taking into consideration the fact that cross-cultural adjustment is a process and not just a state, further longitudinal research is needed. This would greatly contribute to a thorough understanding of the past, present and future of SIEs’ international relocation experiences. In fact, during the second research period (2012–2014) a lack of longitudinal studies could be observed. The conducted studies focused on aspects related to the period before leaving the home country or after arriving in the host country, where participants were asked to think about current aspects or reflect back on their experience.

In order to obtain a more complete understanding of SIEs’ relocation experiences, the multi-informant perspective was introduced in some studies conducted during the second research period, where the spouses’, supervisors’ or host country nationals’ perspective was involved as a complement to SIEs’ view regarding a determinant issue. This complementary perspective is a very important asset of many studies and whenever possible it should be included in future SIE research. In addition, the pre-departure period, which includes expectations, family dynamics, personality and motivations, should be taken into consideration in future studies, since it seems to greatly impact the cross-cultural adjustment process and it has been rarely examined (an exception is Tabor and Milfont 2011).

Another aspect which could be further examined is the conceptual coherence of the SIE concept and its distinction from the other types of mobility patterns. So far, some of the conducted studies during the second research period reviewed the existing literature focused on SIEs. As a result of this, Cerdin and Selmer (2014) proposed four groups of screening questions related to the international relocation (e.g. Have you initiated your expatriation yourself?), regular employment (e.g. Do you have a regular job in the host country?), intention of a temporary stay in the host country (e.g. Was it your original intention to repatriate after a certain time?) and professional qualifications (e.g. Do you have skilled/professional qualifications?). These questions have to be affirmatively answered in order to consider an individual a SIE. Another way of determining if an individual is an SIE is by following the screening criteria proposed by Andresen et al. (2014). They proposed seven demarcation criteria which were claimed to be sufficient for plain differentiation between assigned expatriates, self-initiated expatriates and migrants. However, future research could empirically test these demarcation criteria, as well as the one proposed by Cerdin and Selmer (2014). The sample of such an empirical study should be carefully chosen, in order to guarantee that the different mobility groups (e.g. SIEs, AEs and skilled/unskilled migrants) can be compared. Therefore, some variables should be controlled, such as the participant’s host country and the time spent there. This would contribute to obtaining more precise results, contrary to what happened with some demographic data encountered during the two research periods. In other words, some studies indicate that SIEs are younger than AEs (e.g. Suutari and Brewster 2000; Peiperl et al. 2014) while others do not present any significant results regarding age, gender or marital status (e.g. Froese and Peltokorpi 2013). Most likely these discrepant results are due to the studies being conducted in different contexts, with distinct samples. Therefore, the profile of AEs or SIEs, using variables from the different levels of analysis (micro, meso and macro) should be carefully interpreted, since results across studies may not be cumulative.

Nonetheless, if SIEs would actually prove to be younger than AEs, we should not forget an important age related aspect that could be further explored. We are referring to emerging adults, i.e. individuals aged between 18 and 29 years, who may relocate as SIEs. According to Arnett (2000), during this period individuals prepare for adulthood by undergoing experimentation in love, work and worldview. Many may choose to move abroad in search for these experiences; hence it would be interesting to identify emerging adults’ relocation experiences as SIEs, in terms of their functioning in the new environment, while coping with the challenges associated with the developmental period they are undergoing.

By taking into account similarities and differences between the experiences of expatriates and other mobility groups, an additional aspect which could be explored in future research is expatriate identity. We consider that a more holistic approach to expatriate identity is lacking in the literature, and future studies should explore it because “expatriates who are able to negotiate their identities successfully within the host environment are able to manage the uncertainty associated with that environment more clearly” (Adams and van de Vijver 2015, p. 7).

Besides these suggestions for future research, it should be highlighted that, as mentioned in Table 5, some of the suggestions left by Doherty (2013) were not met so far during the second research period, and they can be taken into consideration in the forthcoming studies. Also, in the table in “Appendix” there is one column dedicated to the authors of some of the reviewed studies, who identified gaps and suggestions for future research. We consider that these are also important suggestions for the evolving knowledge in the area of self-initiated expatriation.

## Conclusion

The identification of the met and unmet suggestions for future research can be seen as a contribution of this paper, since it presents, in a simple way, the conducted research and points out some possible areas of future research intervention. Another contribution of this paper is the updated literature review of the published articles on the self-initiated expatriation topic and systemization of the main findings. This enabled determining that research at different levels of analysis (micro, meso, macro) is rapidly growing and expanding to areas which were previously unexplored. There are new methodologies being used and new contexts chosen for the exploration of the SIE topic. This points out that SIE research is evolving. Although several authors attempted to define what constitutes SIEs and distinguished them from other mobility groups, it is crucial to empirically test the proposed scales. In doing so, sociological and psychological approaches may be adopted, since they are considered to be marginal in the expatriation field, which is dominated by business research (Dabic et al. 2013). Additionally, this rapidly evolving research area would greatly benefit from the conduction of a meta-analysis, since it would complement the present study. In other words, this study compared and contrasted the encountered results in two research periods, while a meta-analysis could combine all the encountered results, identifying patterns and relationships in them.

## Appendix

See Table 6.

**Table 6:** A synthesis of the published studies (2012–2014) focusing on the SIE experience

#	Author(s)	Study focus	Methodology	Findings/main contributions	Gaps identified/future suggestions
1.	Alshammari (2012)	Evaluate the role of two predictors (marital status and previous international experience) on cross-cultural adjustment	237 self-initiated expatriates from public universities in Saudi Arabia filled in an online questionnaire	No significant relation was found between marital status/previous international experience and the three dimensions of cross-cultural adjustment (general, interaction and work adjustment)	
2.	Altman and Baruch (2012)	Explore the factor chance as a motivational driver to undertake expatriation	31 expatriates and repatriates (employees of a major financial institution) were interviewed using a semi-structured interview guide, constructed along the principal stages of the expatriation cycle	A two dimensional (work attractor/motivational driver vs. psychological contract) model is proposed to characterize the main expatriation paths. Corporate self-initiated expatriates identified for the first time	
3.	Beitin (2012)	Explore Syrian SIEs’ experiences and relations with home and host countries	13 Syrian SIE were recruited at the Syrian Expatriate Conference and interviewed about the motives for leaving Syria, their understanding of identity, the relationship with the host country, the factors which helped the transition to the new culture and the relations with the home country	The motives for leaving Syria were related to advance in education and careers. Many men referred the mandate of military service as a factor in leaving. The adjustment issues faced were language barriers and the difficulty of remaining connected to Syria. Relationships with both countries were fluid	Extend this study to the different groups of Syrians and identify the respective differences and similarities
4.	Bergh and Plessis (2012)	Develop and explore a theoretical framework of pre-migration and post-migration career development and success	21 SIE women in the Netherlands, accessed via LinkedIn and snowball sampling, participated in two interactive focus groups	Individual drivers influencing pre-migration and post-migration career development: identity, social support and life phase Factors magnified by SIE women: Identity embeddedness, host country culture, openness to foreigners and existing prejudices and stereotypes against women	Empirical test of the proposed framework
5.	Cao et al. (2012)	1. Provide conceptual clarity by distinguishing SIEs from AEs and skilled migrants 2. Propose a theoretical framework for SIEs’ career success, based on career capital theory	Review of literature in different areas: SIE, expatriation, career studies, migration	SIEs are distinguished from AEs, based on some main results of previous studies (#1, 3, 13, 40): SIEs relocate on their own initiative without any financial support from their employers. They don’t have a definite plan for repatriation; hence their career development is indeterminate Ambiguity exists between migrants and SIEs, differences depending on intrinsic and intangible criteria SIEs’ career success positively influenced by: protean career attitude, career networks, and cultural intelligence. Cultural adjustment mediates these relations, while cultural adjustment acts as a moderator	Expand research besides the influence of macro-contextual factors on SIEs’ career success. Meso-factors (e.g. family, social relationships) could have been taken into consideration
6.	Crowley-Henry (2012)	Explore the existing career metaphors and propose a new one, discuss its implications on future career research and practice	37 SIEs from the Science and Technology Park in South of France were interviewed	The metaphor “river” is considered to more aptly capture the career development of SIEs, by taking into consideration the micro, meso and macro context	Conduct a broader study incorporating the metaphors of this study, along with the nine defined by Inkson (2004, 2007)
7.	Ellis (2012)	Explore and compare the performance management (PM) preferences of SIE New Zealanders in Belgium and host country nationals	10 SIE New Zealanders were recruited through a notice on a website (New Zealanders in Belgium) and 10 Belgians were interviewed	Through content analysis, the following preferences for structured PM of SIEs were found: goal-setting, performance measurement and appraisal, and a performance-based pay component. The Belgians’ preferences for PM incorporate professional and distant relationships. Similarities and differences were found between the two groups, with the differences prevailing	Test the results quantitatively on a larger scale and explore SIEs’ PM preferences before becoming SIEs and after a time in the host country
8.	Froese (2012)	Explore the motivations and cross-cultural adjustment of SIEs in South Korea	30 SIE academics in South Korea were interviewed	Motivational drivers for expatriation: desire for international experience, attractive job conditions, family ties, and poor labor markets in home countries A theoretical framework is proposed between motivational factors and cross-cultural adjustment	
9.	Lo et al. (2012)	Investigate the relationship between job embeddedness (3 factors: HomeCCE, HostCOE, HostCCE) with shocks (unsolicited job offer) and turnover intentions of SIEs in Macau *HomeCCE:* embeddedness towards the organization in which they are employed in the host country *HostCOE:* embeddedness towards the organization in which they are employed in the host country *HostCCE:* embeddedness towards the host country community	127 local staff and 210 SIEs from Macau hotel industry filled out a questionnaire	Factors which may affect the retention of SIE HostCOE mediates the relationship between HomeCCE and turnover intention and willingness to accept unsolicited job offers The following variables moderate this relationship: expatriate-dominated private sector and HostCCE	Longitudinal perspective from job turnover to actual quitting, taking into consideration more workplace shocks
10.	Selmer and Lauring (2012)	Test the motivational drivers found by Richardson and McKenna (2006) and Osland (1995) and their impact on work outcomes (work performance, work effectiveness and job satisfaction)	428 SIE academics from 60 countries employed in 35 universities in 5 northern European countries completed an online questionnaire	Development of a scale for the evaluation of 4 sets of reasons to expatriate: refugee, mercenary, explorer and architect	Incorporate supervisors’ reports while evaluating work outcome variables (e.g. such as work performance and work effectiveness). Longitudinal approach may be used to capture possible changes in the perception of initial behavioral intentions over time. Target SIEs in business firms to test the validity of the findings of this investigation
11.	Shaffer et al. (2012)	Review, summarize and synthetize the empirical research related to the career’s choices, challenges and consequences of different types of global work. Develop a taxonomy of global work experiences and propose an agenda for future research	Literature review of 114 papers	Distinguishes between SIE and AE, based on different criteria Taxonomy of global work experiences (E, SIE, short-term assignees, international business travelers, global domestics, global virtual team members, flexpatriates) based on non-work disruption, cognitive flexibility and physical mobility Leaves some research questions for future research	
12.	Whitman and Isakovic (2012)	Compare the personality traits and stress management/coping strategies between SIEs & AEs	Based on the existing literature, two conceptual models were developed		The empirical test of the proposed framework, will add important knowledge to the existing literature
13.	Yijälä et al. (2012)	Develop and empirically test a model of factors predicting pre-migration adaptation of SIEs	95 SIEs recruited by the European Chemicals Agency in Finland who filled out a questionnaire in the country of departure, while preparing their relocation	European identification, self-esteem and relocation stress act as mediators, while the factors which positively predict for pre-migration adaptation are: previous international work experience, perceived organizational prestige, satisfaction with the time, information and assistance to prepare for the relocation and quality contact with host country nationals during recruitment	Conduct more complex analyses of the pre-migration stage of the relocation process (e.g. longitudinal studies focusing on pre and post-relocation)
14.	Al Ariss and Crowley-Henry (2013)	Critically review how SIEs are different from migrants in the management literature Answer two questions: (1) How are SIEs portrayed compared to migrants? (2) What do we know about SIEs compared to migrants?	Review of 110 peer reviewed articles retrieved from ISI Web of Knowledge database and ABI/Inform. Keywords: “self-initiated expatriation”, “self-directed travel”, “self-initiated foreign experience” and “migration”	Presents how SIEs and migrants are portrayed in the management literature based on: country of origin, gender, education, job position, organizations, period of international mobility, destination countries and description of context Sets out a research framework based on four dimensions: diversity-informed, context specific, reflexive and triangulated methods	
15.	Arp (2013)	Explore foreign executives in local organizations (FELOs) and distinguish them from other types of expatriation (AE and SIE)	46 FELOs from 13 different countries and their host-country peers from organizations founded and headquartered in Malaysia were interviewed	FELOs are different from AEs and can be understood as a rare and specific form of SIEs The content analysis of the interviews identified issues surrounding allegiance, trust, and control, assumptions about income levels, and exposure to heightened local scrutiny as components of the distinct nature of the FELOs’ experience	Explore FELOs in other contexts, since this one was Western to Eastern. Case studies and longitudinal approaches can be used in future studies
16.	Cao et al. (2013)	Empirically test a model of mediation via cross-cultural adjustment between the positive protean career attitude and SIEs’ experience (career satisfaction, intentions to stay and life satisfaction)	132 SIEs in Germany recruited via international platforms (Internations), discussion forums (Toytown Germany) and snowball principles. Path analysis with bootstrap method was used to test the model	Positive cross-cultural adjustment mediates the positive relations between protean career attitude and SIEs’ experienced outcomes (career satisfaction, intentions to stay in the host country and life satisfaction)	Longitudinal research and replication of the study in other destination countries with bigger sample size
17.	Dabic et al. (2013)	Provide a comprehensive literature review on the expatriates and their impact on global business performance	A literature review was conducted and 436 papers were retrieved and analyzed	After 4 decades of research, the existing literature needs further exploration and higher order content	Explore new contexts and organizations; make an effort into systematic approaches
18.	Doherty (2013)	Review and synthesize literature review on the topic of SIE	A thematic analysis was performed on the 49 reviewed published works between 1996 and 2011 on SIE	Constructs of SIE can be analyzed at three different levels of analysis: micro, meso and macro Clarification of the SIE concept	Consider the subjective career experiences; relate individual, micro and meso level variables (e.g. explore how micro level variables can relate to organizational ones, demonstrate whether and how individual career capital can contribute to organizational-level competitive advantage)
19.	Doherty et al. (2013)	Clarify the self-initiated expatriation/expatriate construct	Suddaby’s (2010) elements of construct clarity (definitional clarity, scope conditions, relationships between constructs and coherence) were applied in the clarification process of SIE	A distinction is made between the different types of global work experiences (short-term/flexpatriate, expatriate, organizational SIE, SIE, AE, international students and migration) based on the criteria: initiation, goals, funding, focus, career impetus, intended duration, employment and occupational category)	
20.	Froese and Peltokorpi (2013)	Explore how AEs and SIEs differ in terms of individual factors, job-related factors and expatriate outcomes. Determine which individual or related factors can explain the difference in expatriate outcomes between AEs and SIEs	124 SIEs and 57 AEs from 25 different countries and living in the Tokyo area were recruited through numerous intermediaries (e.g. chambers of commerce, alumni associations). They filled out a questionnaire	*Individual factors*: significant differences in Japanese language proficiency and overseas experience with SIE presenting higher levels; no significant differences in age, gender, marital status or education; *Job related factors:* SIEs worked more under Japanese supervisors (acts as a mediator on their job satisfaction) and domestic companies, whereas AEs worked for foreign companies and reported higher levels of *job satisfaction* than SIEs *Cross*-*cultural adjustment:* SIEs were more adjusted to interacting with host country nationals, because of their longer stay in the host country and higher host-country language proficiency (mediators)	Test the relations in non- Asian contexts. Explore further the role of family since this study only took into consideration marital status. Host country nationals’ perspective could also be explored along with the SIEs’ work performance
21.	Guo et al. (2013)	Examine the career experiences of repatriated SIEs	20 repatriated Chinese SIEs were interviewed about their motivations to repatriate and the international experience	Career agency is impacted by both individual (e.g. personal control, proactivity, self-determination) and contextual factors, which provide support for Tams and Arthur’s (2010) six dimensions of career agency	
22.	Isakovic and Whitman (2013)	Explore the adjustment of SIEs, using Black et al.s’ (1991) model as the theoretical foundation. Test the influence of previous overseas work experience, foreign language ability and culture novelty on adjustment	An online survey was filled in by 297 academic SIEs working in ten higher education institutions in United Arab Emirates	Previous overseas experience has a positive relationship with SIEs’ adjustment, while culture novelty has a negative one. Contrary to what was predicted, foreign language ability was not positively related to adjustment	Examine the other factors included in Black et al. (1991) in order to determine if they apply to SIEs and how they differ from AEs
23.	Lauring and Selmer (2013)	Compare public and private sector SIEs, regarding work outcomes (degree of performance, effectiveness and job satisfaction) and the effect of creativity on work performance	329 SIEs residing in Denmark (119 private sector and 210 public sector) filled in an online questionnaire. They were recruited through an association for international residents in Denmark	On average, SIEs in the public sector present a higher degree of performance and effectiveness than SIEs in the private sector. Contrary to what was predicted, this doesn’t happen with job satisfaction. For SIEs in the private sector vs. public sector, there is a stronger positive association between creativity and performance, creativity and effectiveness, but not between creativity and job satisfaction	Use more homogenous samples of SIEs. Rely on information from supervisors and colleagues. Distinguish between incremental and radical creativity since literature indicated that incremental creativity is more present in the public sector while radical creativity is more often found in the private sector
24.	Scurry et al. (2013)	Examine how SIEs position themselves in terms of identity	20 SIEs working in a Qatari public shareholding company were interviewed about their motives behind becoming an SIE, work and life experiences in Qatar and the organization, career paths, and relationships with others	The narratives of self were framed within structural constrains and patterns of adaptation. As part of these themes two narratives were identified: narrative of mobility (ambiguity in relation to temporal and spatial parameters of adaptation) and opportunity (motives behind becoming a SIE centered on opportunity)	More empirical work to map the experiences of SIEs. Explore experiences of female SIEs in a male dominated working environment
25.	Showail et al. (2013)	Empirically test the relation between role ambiguity, organization identification (mediator) and job performance, being moderated by information seeking about the organization and perceived organizational support	A multi-informant perspective was used in the completion of the questionnaire. 138 supervisors and 154 SIEs were recruited from six Saudi Arabian companies employing foreign employees	The relationship between role ambiguity and job performance was significant. This relationship was mediated by organizational identification and moderated by information seeking and perceived organizational support	Explore the workers’ motivations for engaging in the identified behaviors
26.	Tharenou (2013)	Examine scholars’ proposal that SIEs are an alternative to AEs for filling key positions at lower cost	21 empirical studies were analyzed, comparing SIEs, OEs and MNC locals in terms of interaction with the local community; employing organization and work interactions; managerial roles and skills; attachment to employer; and attachment to living and working abroad	Situations where SIEs can replace AEs: roles requiring cross- cultural and host location-specific competencies and generic competencies. Along with MNC locals, SIEs can replace AEs in managing local, host-country culture within a subsidiary and dealing with local environment. At a lower cost SIEs can replace AEs for filling specialist professional and lower, middle management roles. They cannot replace AEs for roles requiring firm-specific competencies	Determine to what extent the competences developed are similar among SIEs, AEs and local employees who work a comparable, lengthy time in MNCs. Explore if the amount of international experience can moderate the relation between competences and the suitability of SIEs replacing AEs
27.	Thorn et al. (2013)	Explore the relation between motives and mobility patterns	2608 SIE New Zealanders were recruited through university alumni organizations, professional associations and snowball technique. They were asked to fill out an online questionnaire	The identified motives were cultural and travel opportunities, career, economics, affiliations, political environment, and quality of life. The mobility patterns were evaluated in terms of frequency, duration and cessation of mobility and the nature of the destination in terms of development level and cultural distance. CHAID analysis was used to test for the motivation predictors of mobility patterns. Desire for cultural and travel opportunities was the best predictor of cessation of mobility and development level in the host country. Career motives predicted duration of mobility and cultural difference of the destination	Determine if return propensity (an imminent return to the home country) or other current mobility intentions can be predicted by motives for previous mobility. Explore the development of mobility motivations overtime (longitudinal study)
28.	Andresen et al. (2014)	Clarify the distinctions and develop a framework of different types of self-initiated and assigned expatriates	Literature review of 136 articles from sociology, psychology and economics. Content analysis and the Rubicon model are used to analyze data	AEs, SIEs and migrants are distinguished on individual and organizational levels based on different criteria; a decision tree is proposed in order to distinguish between AEs, inter- self-initiated expatriates, intra-self-initiated expatriates and drawn expatriates: (1) Move from one geographical point to another via crossing national borders (yes/no) (2) Change of dominant place of residence which is the center of a person’s life (yes/no) (3) Execution of work in the form of dependent or independent employment (yes/no) (4) Legality of employment (legal vs illegal) (5) Initiator of key binding activity in job search (organization vs individual) (6) Work contract partner (current vs new)	Empirical proof of the proposed decision tree and add more demarcation criterions (e.g. based on motive)
29.	Bjerregaard (2014)	Examine the interaction between institutional contexts and agency in self-initiated global careers	38 SIEs and 11 spouses were interviewed. Observational notes were taken and documents were analyzed	Since institutional interventions can form SIEs’ careers and work experiences, the results of this study show how public institutions shape international experiences within and outside the workplace. More precisely, the public institutions ease, drive and support the global career mobility and agency of SIEs	Find how institutions of host countries at different levels are engaged in international professionals’ encounters with them, from the programs and policies of central ministries to the frontlines of service delivering and people processing organizations (cf Hasenfeld 1972)
30.	Cao et al. (2014)	Explore SIEs in organizational context, specifically through a moderation and mediation model: the effect of perceived organizational support (POS) on SIEs’ intention to stay in the host country, is moderated by career networks of host and home country nationals and mediated by career satisfaction	112 SIE employees in Germany were recruited through numerous intermediaries (e.g. Internations, Xing, ToytownGermany) and filled out an online questionnaire	A direct positive effect between POS and intention to stay was found. However, there was a significant negative indirect effect between POS and intention to stay when the career network of home country nationals was large. POS has a positive effect on SIEs’ career satisfaction and intention to stay in the host country	Explore the impact of family support and supportive immigration/re-emigration policies. Determine what contributes to SIEs’ POS and explore other outcome variables (e.g. turnover intentions)
31.	Cerdin and Le Pargneux (2014)	Explore how individual career characteristics (protean career attitude, boundaryless career attitude, careerist attitude and career fit) influence the success of international mobility (job satisfaction, career satisfaction, intention to leave)	303 French SIEs and AEs working in 57 countries were recruited through companies, professional associations and snowball sampling. They were asked to fill in an online questionnaire	Careerist attitude and career fit explain international mobility success, while the influence of protean and boundaryless career attitude is not very clear Careerist orientation is the individual career characteristic which better explains international mobility success	Collect data on expatriate performance and refine the success criteria of international mobility, by focusing on the accomplishment of organizational tasks and/or the achievement of key organizational objectives
32.	Cerdin and Selmer (2014)	Contribute to the conceptual clarity of SIE (provide definition and criteria which distinguishes SIE from other types of mobility patterns)	Analysis of several articles, in order to determine how SIEs are defined	SIEs are defined as ‘expatriates who self-initiate their international relocation, with the intentions of regular employment and temporary stay, and with skills/professional qualifications’. Operationalization of SIE is proposed based on dichotomous questions based on four criteria: self-initiated international relocation, regular employment (intentions), intentions of a temporary stay, skilled/professional qualifications	Use the proposed criteria to screen the sample of future studies. Explore the HRM practices of the hiring organizations of SIEs
33.	Farndale et al. (2014)	Explore Global Talent Management strategies of how individual and organizational goals can be balanced for expatriation assignments	Two case studies realized in multinational corporations, with interviews and observations conducted with10 Global Mobility/Human Resources specialists and 6 expatriates	Results demonstrate a largely financially driven balancing act between self-initiated and organizational assigned expatriate assignments	7 research propositions are left for future research
34.	Lauring and Selmer (2014)	Examine whether inherent demographic characteristics (age/gender) and acquired demographic characteristics (marital status/seniority) differentiated work outcomes (job adjustment, time to proficiency, performance and job satisfaction). Associations between these variables and global mobility orientation were also explored	640 academic SIEs residing in Greater China recruited through LinkedIn profiles and university web pages were asked to fill in an online questionnaire. Full/Chair Professors and Associate Professors made up the senior group	Female SIEs have better job performance than male SIEs. Married SIEs have better time to proficiency and job performance than unmarried SIEs. Senior SIEs have better job performance and satisfaction than junior SIEs. Profile of successful SIEs in Greater China: female, married and occupying a senior position. For males and younger individuals, there was a stronger relationship between global mobility orientation and work outcomes	
35.	Lauring et al. (2014)	Explore the influence of demographic characteristics (age, marital status, nationality, past relocation experience) on tourism-oriented motivation (seeking reasons, escape reasons) and work-related motivation (career & financial reasons)	428 academic SIEs from 60 different countries working at 34 different universities located in 5 northern European countries	Tourism-oriented and work-related motivations were stronger among academic SIE who are younger, non-married, non-EU and with short experience. Non-EU SIEs arriving in the EU have stronger financial and seeking motivations	Expand this study to other types of SIEs and their respective partners. Conduct qualitative research to better explain how temporary work relocations may connect to tourism motivation
36.	Muir et al. (2014)	Determine what factors influence professional women to self-initiate their expatriation, how do their careers look like and how do they construct their career experiences	25 SIE western women working and living in Beijing were interviewed	The motivations, career types and patterns of SIE women are complex and varied. 4 career patterns are identified: reinventors, reinvigorators, reversers and rejecters	Gather data from SIE women in different host countries. Longitudinal studies and analyze data using discourse analysis. Gender explored if it acts as an inhibitor or facilitator
37.	Nolan and Morley (2014)	Examine the relationship between person-environment fit (person-job needs-supplies fit, person-job demands abilities fit, person-supervisor fit) and cross-cultural adjustment	369 SIE doctors working in the public sector hospitals of Ireland were recruited, after the directors’ approval	The dimensions of person-environment fit had different effects on cross-cultural adjustment. Person-job needs-supplies fit had no relationship	
38.	Peiperl et al. (2014)	Analyze the trends and mobility patterns of AEs and SIEs	55,915 highly skilled individuals who crossed borders between 1990 and 2006	Results show a steady increase in domestic job mobility over the studied period, with cross-border mobility keeping pace. SIEs and AEs have become shorter term, with AEs more likely to move to peripheral economies than SIEs. AEs may be on the decline in the EU context as compared with SIEs	
39.	Richardson and McKenna (2014)	Investigates the role of networks during expatriation (their impact in the expatriation engagement, how are they formed and maintained, what are their benefits)	51 organizational SIEs working in a professional services firm (Mintech) in Canada. Methodological pluralism (documentary analysis-company expatriation protocol, interviews with 2 HR managers and expatriated workers)	The role of networks are important for organizational SIEs, because they play an instrumental role in fulfilling their desire to expatriate. While being in the host country, they intend to develop, expand and consolidate their ties and networks, since these contribute to the integration and performance of the global business	Explore more structured relations between organizational culture, HRM processes, organizational SIEs and the development of ties and networks
40.	Rodriguez and Scurry (2014)	Explore the career capital development of SIEs in Qatar	20 SIEs working in a Qatari public shareholding company, were interviewed about their social and work experiences	The dynamics at micro, macro and meso levels influence the development of SIEs’ career capital, in the following three domains: SIE as cosmopolitan globetrotters, SIE as experts, SIE as outsiders	
41.	Selmer and Lauring (2014a)	Explore the effect of the personality traits dispositional affectivity on the adjustment of SIEs	329 SIEs in Denmark were asked to fill in an online questionnaire	Beneficial associations between positive affectivity and adjustment	
42.	Selmer and Lauring (2014b)	Explore the adjustment of self-initiated expatriate academics, comparing adult third-culture kids with adult mono-culture kids	267 academic SIEs in Hong Kong filled in an online questionnaire	Adult third-culture kids have a greater extent of general adjustment, but not interaction or work adjustment	
43.	Supangco and Mayhofer (2014)	Determine what factors affect work-role transition outcomes and how the type of expatriation influences work role transition outcomes	106 Filipinos working in local and multinational organizations in Singapore filled in an online questionnaire	Work adjustment is explained by self-efficacy beliefs of the global employees. Job satisfaction is explained by job factors (role discretion and role conflict) and organizational or job context factors (supervisory support and perceived organizational support). Both work role adjustment and job satisfaction are not influenced by whether or not the global employee is company assigned or self-initiated	
44.	Vance and McNulty (2014)	Determine similarities and differences across gender	45 American expatriates in five Western and Central European countries were interviewed	The results support Vance’s (2005) career development model	
45.	von Borell de Araujo et al. (2014)	Understand the challenges and strategies used by SIEs and AEs in adjusting to the Brazilian culture	Semi-structured interviews were conducted with 20 AEs and 24 SIEs	Challenges: foreignism, formalism, personalism and jeitinho. The strategies used to overcome these challenges differ between SIEs and AEs. The SIEs were less critical and more willing to follow typical Brazilian behavior for resolving problems related to adjustment	Explore the encountered challenges in other host countries characterized by informal influence processes (e.g. Chinese guanxi, Russian svyazi, Arabian wasta)
